# Molecular prevalence of *Coenurus cerebralis* in sheep exhibiting neurological symptoms in Kazakhstan

**DOI:** 10.3389/fvets.2025.1620425

**Published:** 2025-07-01

**Authors:** Aigerim Kozhayeva, Abzal Kereyev, Alexandr Shevtsov, Bolat Abdigulov, Darkhan Smagulov, Saltanat Khamzina, Botagoz Kulzhanova, Kulsara Nurzhanova, Lyaila Bupebayeva, Aigerim Khamzina

**Affiliations:** ^1^Research School of Veterinary Medicine and Agriculture, Shakarim University, Semey, Kazakhstan; ^2^Institute of Veterinary and Agrotechnology, Zhangir Khan West Kazakhstan Agrarian Technical University, Uralsk, Kazakhstan; ^3^Laboratory of Applied Genetics, National Center for Biotechnology, Astana, Kazakhstan; ^4^Agricultural Innovation and Technology Park, West Kazakhstan Innovation and Technological University, Uralsk, Kazakhstan; ^5^LLP Scientific and Educational Center “Qazyna”, Almaty, Kazakhstan; ^6^Green Biotechnology and Cell Engineering Laboratory, Kazakh National Agrarian Research University, Almaty, Kazakhstan

**Keywords:** *Coenurus cerebralis*, Kazakhstani sheep, PCR, sequencing, clinical, phylogenetic analysis

## Abstract

Coenurosis, caused by the larval stage of *Taenia multiceps* — *Coenurus cerebralis*, is a severe parasitic disease that affects the central nervous system of sheep and causes significant economic losses in livestock farming. This study aimed to determine the molecular prevalence of *Coenurus cerebralis* in sheep with neurological symptoms in Kazakhstan using conventional PCR followed by Sanger sequencing. The study was conducted in the West Kazakhstan and Abay regions. However, insufficient data from other regions limits the development of effective national control measures. Of the 100 sheep examined, clinical signs of the disease were detected in 15 animals. During molecular analysis of the mitochondrial genes *COX1* and *NAD1*, positive results were obtained in 9 samples (81%). The data obtained confirm the high diagnostic value of PCR and the effectiveness of mitochondrial markers for identifying *C. cerebralis*. The results contribute to a better understanding of the epidemiology and pathogenesis of cenurosis and can be used to develop effective strategies for the prevention and control of the disease in regions with developed sheep farming. Kazakhstani isolates are grouped within a single haplotype (Hap47), which belongs to a major, widely distributed lineage. This suggests potential links with other countries and may reflect historical migration patterns or species dispersal routes. Isolates from Italy, Turkey, and Iran also show a strong association with the principal haplotype lineages, indicating a shared genetic background.

## Introduction

*Coenurus cerebralis (C. cerebralis)*, the larval stage of the cestode *Taenia multiceps*, is the causative agent of cerebral coenurosis. This parasitic disease affects the central nervous system in sheep and other ruminants. Coenurosis is a serious health problem for sheep, especially in regions such as Kazakhstan, where livestock farming is widespread. This parasitic infection primarily affects the central nervous system of sheep, leading to severe neurological symptoms and significant economic losses in the agricultural sector (1, 2). The disease manifests when sheep ingest eggs from definitive hosts, usually canids, through contaminated feed or water. Once inside the body, the oncospheres migrate to the brain, where they develop into cysts called cysts, which can grow large enough to exert significant pressure on nerve tissue, leading to clinical signs such as ataxia, convulsions, and even death (3, 4).

The prevalence and molecular characteristics of *C. cerebralis* in sheep with neurological symptoms are the subject of growing research interest. Studies have shown that infection rates can vary significantly depending on factors such as geographical location, farming practices, and host immunity (4, 5). In Kazakhstan, understanding the molecular epidemiology of *C. cerebralis* is crucial for developing effective control strategies. Recent efforts to characterize the molecule have revealed genetic variations among isolates that may influence the pathogen’s virulence and transmission dynamics (1, 6). In addition, the use of advanced molecular techniques such as PCR and transcriptomic analysis has provided insight into the genetic structure of the parasite, helping to identify specific strains that may be more virulent or resistant to treatment (3, 7). Molecular studies using PCR have been carried out in countries such as Turkey (8), Pakistan (9), China (10), Saudi Arabia (11), Jordan (12), Ethiopia (13), Iraq (14), and Iran (15) to detect and genetically characterize *Taenia multiceps* isolates.

The impact of coenurosis extends beyond the health of individual animals, affecting herd productivity and the economic viability of farmers. This disease causes direct losses through mortality and reduced productivity and creates problems for disease management and control in livestock populations (2, 16). Therefore, comprehensive studies investigating the molecular prevalence of *C. cerebralis* in sheep with neurological symptoms must inform Kazakhstan’s public health and veterinary practices. By identifying this parasite’s genetic diversity and epidemiological patterns, stakeholders will be able to develop more effective strategies to mitigate its impact on sheep farming and preserve animal health. Sheep farming is an essential sector of animal husbandry in Kazakhstan. It plays a significant role in the country’s economy, providing meat, milk, wool, and hides. However, this lack of regional data makes it impossible to obtain a complete picture of the true prevalence of the disease across the country. Our study was conducted to compare two distinct regions for parasite prevalence in order to assess ecological plasticity.

## Materials and methods

### Sample collection

**Figure 1:**
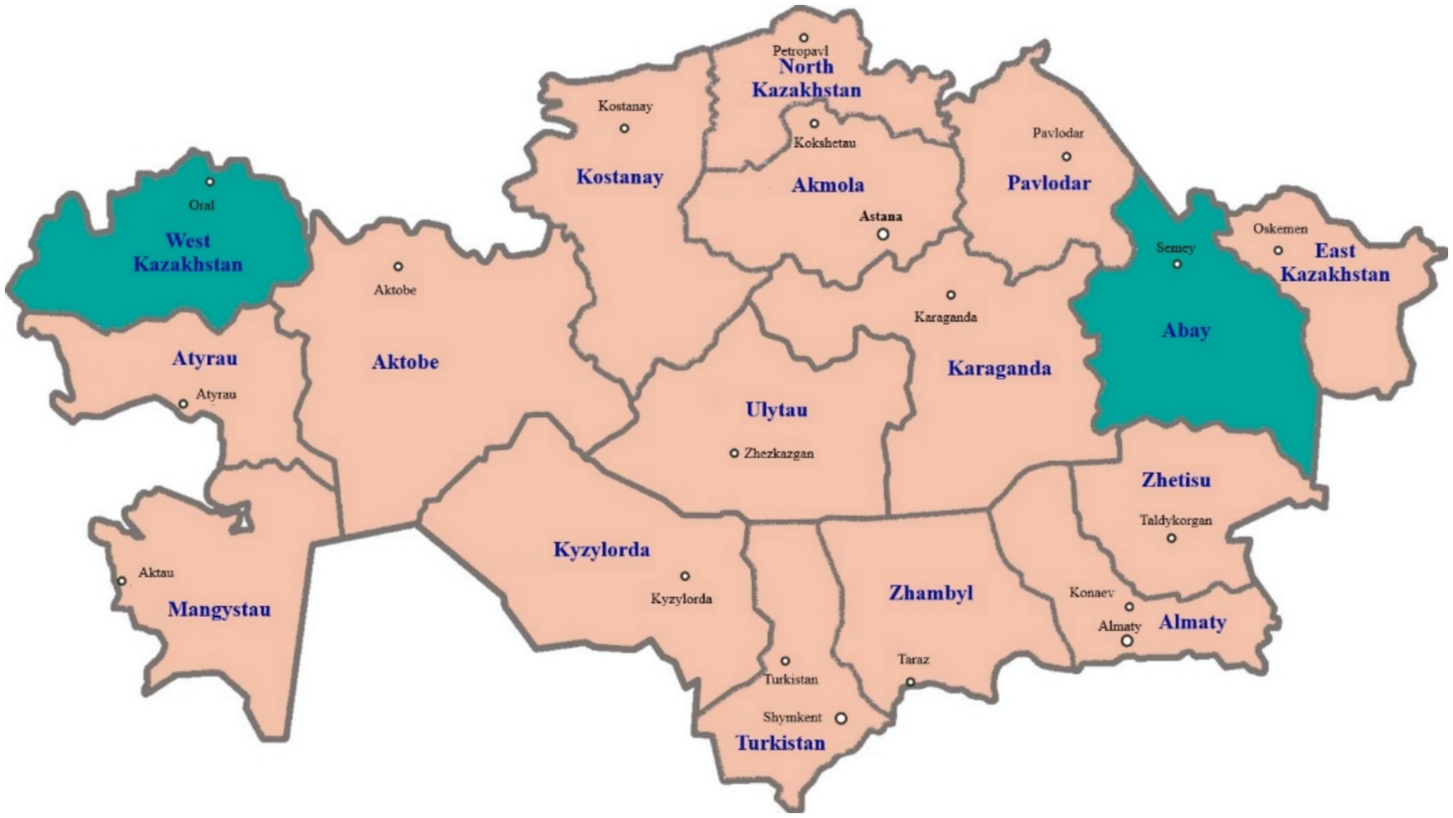
Map of Kazakhstan showing the two regions West Kazakhstan region (Oral city) and Abay region (Semey city) where the examined sheep were sampled in 2024.

The scientific research was conducted in 2024 year in the West Kazakhstan region, in the village of Atameken, Taskala district, where the Akzhaiyk sheep breed (50 heads) is raised, and at the “Ebetey” farm in the Zhanasemei district of the Abay region (East Kazakhstan), where the Kazakh fat-tailed coarse-wool breed (50 heads) is bred (Figure 1). In 2024, 100 sheep were delivered to a slaughterhouse in Oral city of West Kazakhstan and Semey city of the East Kazakhstan.

Of the 50 animals sampled from each region, 15 were under 1 year of age, 15 were between 1 and 3 years, and the remaining 20 were over 3 years old, with an equal sex distribution of 25 females and 25 males in each group. For the molecular characterization, cyst material was collected from the brain tissue and fixed in 70% ethanol untiluse. Genomic DNA extraction was performed from both the protoscoleces and cyst walls using the Wizard Genomic DNA Purification Kit (Promega, USA; Cat. No. A1125) according to the manufacturer’s protocol from 20 samples.

### PCR

For PCR analysis, 11 scolex samples were selected. Target regions of the mitochondrial genes *COX1* and *NAD1* were amplified using two pairs of primers: JB3: 5′-TTTTTTGGGCATCCTGAGGTTTAT-3′, JB4.5: 5′-TAAAGAAAGAACATAATGAAAATG-3′ and JB11: 5′-AGATTCGTAAGGGGCCTAATA-3′, JB12: 5′-ACCACTAACT AATTCACTTTC-3′, respectively. The polymerase chain reaction consisted of an initial denaturation at 95°C for 5 min, followed by 40 amplification cycles, each including denaturation at 94°C for 45 s, primer annealing at 51°C (for *COX1*) and 55°C (for *NAD1*), and elongation at 72°C for 45 s. Final elongation at 72°C lasted 10 min and was required for complete amplification of all fragments. Amplification was performed using a SimpliAmp thermal cycler (Thermo Fisher Scientific, USA) in a total volume of 25 μL. The reaction mixture included: 10 pmol of each primer of the respective pairs, 10 mM Tris–HCl (pH 9.0 at 25°C), 50 mM KCl, 2.5 mM MgCl₂, Triton X-100 at a final concentration of 0.1%, dNTPs at 0.2 mM each, 1.5 units of HS Taq polymerase (Biolabmix, Russia), and 5 μL of DNA. PCR results were visualized by fragment separation on a 1.5% agarose gel in 1x TAE buffer using the intercalating dye ethidium bromide. A Step 100 Long DNA marker (S-8103, Biolabmix, Russia) was used as a molecular weight marker. Electrophoresis was performed using a horizontal gel electrophoresis chamber, Cell Model 192 (Bio-Rad, USA) and a power supply EPS 601 (GE Healthcare / Amersham Pharmacia, China). Results were documented using the Gel Doc XR + system (Bio-Rad, USA) and Quantity One software (Bio-Rad, USA).

### PCR sequencing and phylogenetic analysis

PCR sequencing was performed using the BigDye™ Terminator v3.1 Cycle Sequencing Kit (Applied Biosystems, Thermo Fisher Scientific, USA) according to the manufacturer’s instructions. The purified DNA was resuspended in 14 μL of formamide and after denaturation for 5 min at 95C, followed by separation of fragments on an automatic genetic analyzer 3730xl DNA Analyzer (AppliedBiosystems). The nucleotide sequences obtained using the forward and reverse primers were analyzed and combined into a common sequence using SeqMan software (Lasergene, DNASTAR). The obtained nucleotide sequences were identified relative to the available nucleotide sequences deposited in the GeneBank databases (www.ncbi.nih.gov) using the BLAST algorithm. BioNumerics 8.1 software (Applied Maths NV, Belgium) was used to analyze haplotype diversity and visualize the relationships between sequences.

## Results

### Necropsy findings

This study established an overall prevalence of 9% for *C. cerebralis* in sheep, based on PCR and sequencing results of brain tissue samples collected from two regions of Kazakhstan. These animals were physically examined for clinical signs of coenurosis. After slaughter, the heads of selected sheep were collected and delivered to a laboratory in a cold chain for necropsy and collection of PCR analysis samples. The skull samples were dissected using an electric saw, and the meninges were incised with a scalpel blade. The exposed brains were thoroughly examined for gross pathological lesions, size, location, and number of cysts in each brain region. Of the 100 sheep, 22 samples showed clinical signs of coenurosis and were selected for inclusion in this study. Clinical prevalence may differ significantly from necropsy results: not all sheep with clinical neurological signs had cysts in the brain. Of the 22 sheep examined, 15 showed various neurological signs, such as head tilt, ear drooping, circular movements, blindness, pressing the head against the surface, and lying down. A study of 15 sheep with neurological signs showed that 15 individuals tested positive (9 Akzhaiyk breed and 6 Kazakh fat-tailed coarse-wool breed). During clinical examination, body temperature, respiratory rate, and pulse were within normal physiological limits. The cysts comprised transparent hyaline membranes with various internal protoscolexes immersed in a translucent fluid. The number of scolexes in one cyst varied from 10 to less than 100.

**Figure 2:**
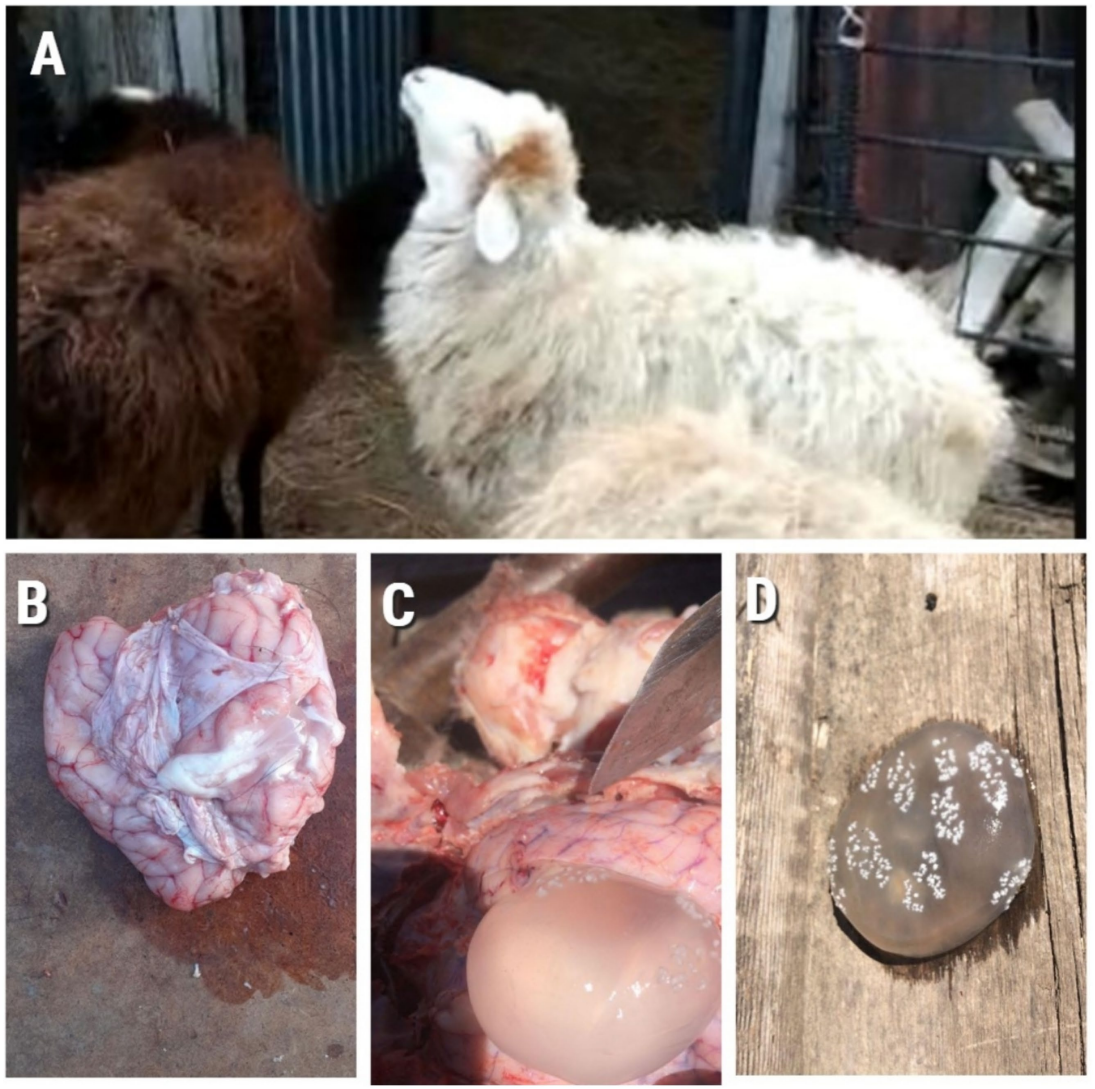
**(A)** Animal showing clinical signs of Coenurosis; **(B)** Sheep brain at autopsy; **(C)** and **(D)** Cyst formation.

The results of this study showed that 15/100 animals demonstrated clinical signs of coenurosis, including ataxia, circular movements, head pressing, convulsions, anorexia, and lethargy. The necropsy results of these 15 sheep heads indicated the presence of cysts in 15 brains. These cysts were oval or spherical and varied in size. They were covered with a thin, fragile membrane and filled with a translucent fluid (Figure 2).

### PCR results and phylogenetic analysis

**Figure 3:**
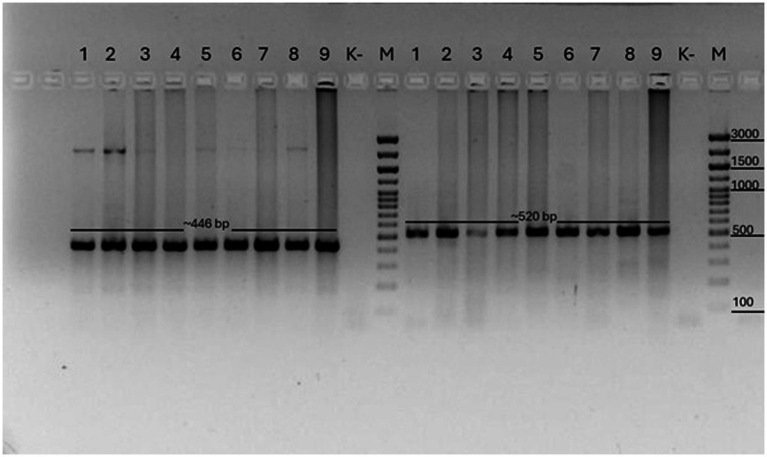
COX1 PCR results of *Coenurus cerebralis* cysts. M: Marker (446 bp) and NAD1 results of *Coenurus cerebralis* cysts. M: Marker (520 bp). Legend: (1–9) samples, numbered according to base pair size; (M) molecular weight marker (Biolabmix) (100–3,000 bp); (K–) negative control sample.

PCR analysis of this study showed that 9/15 (81%) samples were positive for *C. cerebralis* in these animals (Figure 3).

As a result of amplification of the COX1 gene, a specific PCR product of 446 base pairs was obtained in all the studied samples (1–9). Fragments of the expected length of 520 base pairs were also obtained during amplification of the NAD1 gene. The amplification products were clearly visualized on the electrophoregrams, which indicates the good quality of the isolated DNA and the specificity of the primers used. The samples are designated by numbers (1–9) in accordance with the order of their analysis. Control samples included a molecular marker (M) (Biolabmix, range 100–3,000 bp) to determine the fragment sizes and a negative control (K–) that did not contain a DNA template. There was no amplification in the control sample, which excludes the possibility of contamination.

Identification of amplicons corresponding to fragments of *Taenia multiceps* mitochondrial DNA confirms that the studied cysts belong to the larval stage of this parasite — *C. cerebralis*, the causative agent of coenurosis. The obtained results demonstrate the high efficiency of using mitochondrial markers COX1 and NAD1 for rapid and reliable molecular identification of the causative agent of coenurosis in farm animals.

**Figure 4:**
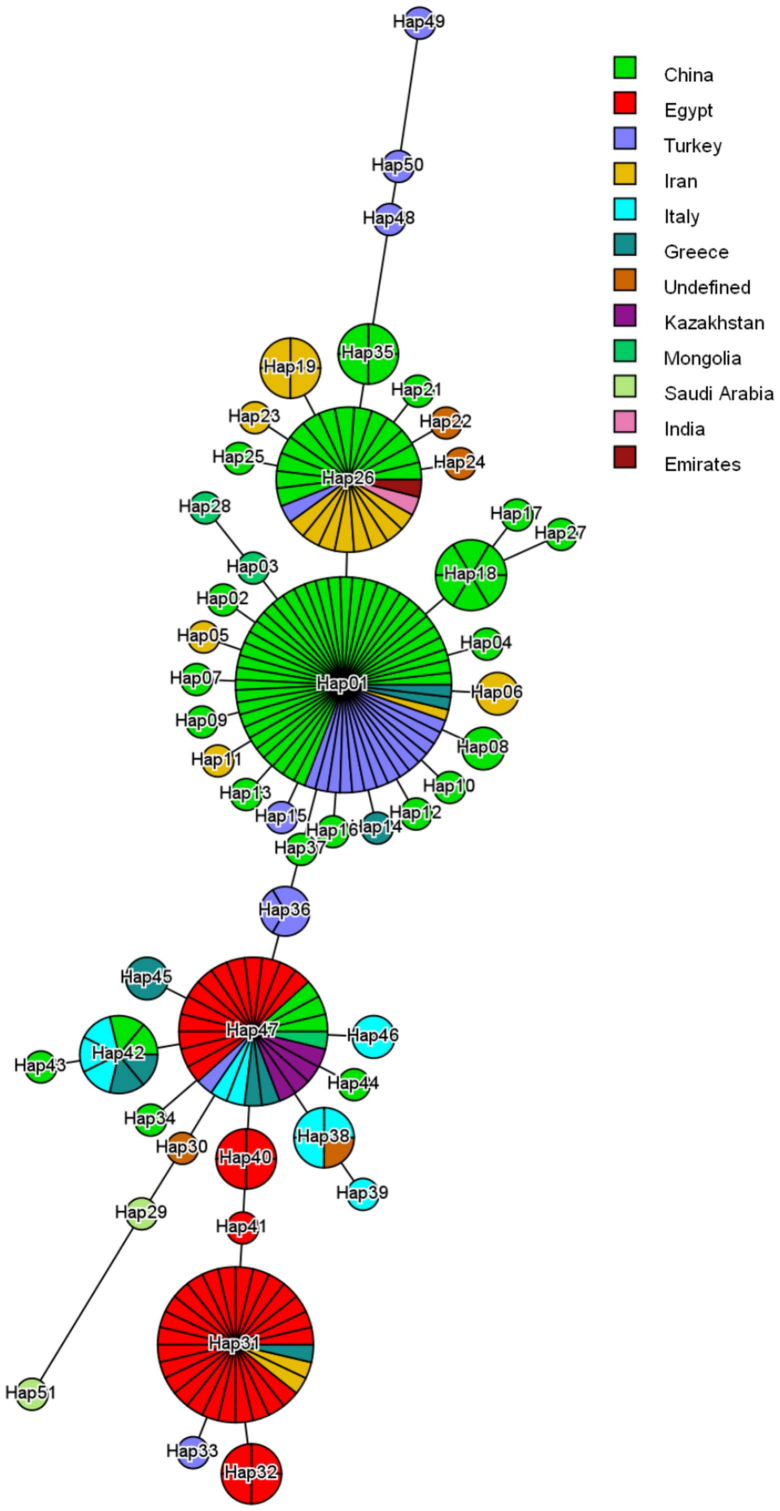
Haplotype network constructed from partial cox1 nucleotide regions of *Taenia multiceps* isolates worldwide. The network describes the distribution of identified haplotypes (Hap1-51) with respect to the country of origin, which is indicated by different colors. The size of the circle corresponds to the haplotype frequency. The number of mutations that distinguish haplotypes is shown by dashed marks.

The sequences used in this study have been deposited in the NCBI GenBank database under the following accession numbers: PP907960.1 – PP907963.1. Four major haplotypes were observed (Figure 4). Hap01 was the most common and involved 55 isolates. Hap01 and their adjoining minor haplotypes circulate in China, Turkey and Iran; however, some Greece isolates were included. This node (Hap01) is possibly one of the central nodes of origin. On the contrary, the three other major haplotypes Hap26, Hap47 and Hap31 involved 27, 28 and 33, respectively. Hap26 and their minor haplotypes circulate mostly in China and Iran, some of the isolates in India, Turkey and Emirates. Hap 47 and their minor haplotypes focusing mostly on Egypt, China and Kazakhstan, some of the isolates in India, Turkey and Emirates. Hap31 and their minor haplotypes detected in Egypt, by several isolates circulate in Iran and Greece. Hap47 and Hap36 are more diverse than the Hap31, particularly within the Egyptian haplotype, which shows about local evolution.

## Discussion

PCR analysis has proven valuable in diagnosing cenurosis and related parasitic conditions (17). Similar to the findings of Gazioglu et al. (17), who successfully identified *Taenia multiceps* in sheep and calves with coenurosis with CO1-PCR assay, yielding a 446 bp band (17), our study also detected a specific 446 bp fragment in all positive samples. This confirms the reliability and consistency of the COX1 gene as a molecular marker for the identification of *T. multiceps* in clinical specimens. For neurocysticercosis, a PCR assay in cerebrospinal fluid (CSF) demonstrated 72.2% sensitivity and 100% specificity, with particularly high sensitivity (90.9%) for extraparenchymal cases (18). Another real-time PCR assay on CSF samples achieved 83.3% detection rate and 100% specificity in neurocysticercosis patients (19).

In our study, animals were pre-selected based on clinical signs consistent with coenurosis, including head tilting, circling, ataxia, and signs of depression. These symptoms align with those described in previous reports, such as altered head carriage and circling movements (17, 20). Molecular identification using PCR and sequence analysis of mitochondrial genes (CO1, 12S rRNA) confirms the presence of *Taenia multiceps* (17, 20). Interestingly, genetic analysis of cerebral (*C. cerebralis*) and non-cerebral (*Coenurus gaigeri*) forms shows 100% identity based on the enolase gene and mitochondrial markers (cox1 and nad1), suggesting that *T. gaigeri* may not be distinct from *T. multiceps* (21). Molecular prevalence of *C. cerebralis* in sheep from Igdır showed that out of 300 sheep with clinical signs, cysts were found in 246 brains. PCR with COX1 gene amplification confirmed 243 cases of infection. Sequencing showed 99.19–100% similarity with known isolates. Infection was more common in young males and in winter, highlighting the high prevalence of coenurosis in the region (8).

Our study found that 9 out of 100 sheep from two different ecological zones of Kazakhstan (steppe and arid West Kazakhstan, and mountainous and forested East Kazakhstan) were positive for coenurosis by PCR. Interestingly, despite the harsher climate in the West, more cases of infection were recorded in this region. This may indicate that environmental factors such as overgrazing, lack of clean water, and animal movement patterns can unpredictably influence the spread of parasites. The findings highlight the importance of conducting more in-depth environmental and epidemiological studies in different zones of Kazakhstan. These observations are in line with preliminary conclusions made by Kushaliev in 2023, who also suggested a potential link between ecological stressors and the prevalence of coenurosis in certain regions (22). We found that 8 out of 9 positive cases were from sheep aged between 1 and 3 years, further supporting previous studies that report a higher prevalence of *C. cerebralis* in younger animals (12–15). Wild animals share some haplotypes with domestic sheep and goats. For example, the fox and mouflon isolate from Italy were assigned to the Hap47 haplotype, which is also widespread among animals in that country (23). Similarly, the Chinese yak isolate fell into the Hap35 haplotype, common to sheep and goats from China. In the case of cattle, the Italian samples included three haplotypes (Hap42, Hap46, and Hap47) that are also found in Italian sheep. However, three unique haplotypes (Hap48, Hap49, and Hap50) found in Turkish cattle are thought to represent a distinct genetic lineage (23).

Our findings demonstrate that the Kazakhstani isolates belong to the widespread haplotype Hap47, which is also found in various domestic and wild animals across several countries, including Italy. This suggests the existence of a common genetic lineage possibly maintained through historical patterns of animal movement and trade. The presence of shared haplotypes, such as Hap47, among different species (e.g., fox, mouflon, sheep, and cattle) highlights the potential for cross-species transmission and supports the idea of a complex epidemiological network. Furthermore, the detection of unique haplotypes (Hap48–50) in Turkish cattle points to regional diversification and potentially distinct evolutionary pathways. Overall, these results underline the importance of integrating genetic data with ecological and historical context to better understand parasite transmission dynamics and population structure on a broader geographic scale.

## Conclusion

The study established the molecular prevalence of *C. cerebralis* among sheep with neurological symptoms in Kazakhstan. PCR analysis with amplification of the mitochondrial genes *COX1* and *NAD1* demonstrated high efficiency in detecting the larval stage of *Taenia multiceps*. Of the 15 animals with clinical signs of coenurosis, 9 (81%) tested positive by PCR, confirming the presence of the parasite and the high reliability of the molecular markers used. This suggests that not all animals displaying neurological symptoms are affected by coenurosis; other conditions or infections may be responsible. Kazakhstani isolates fall under a single, widely distributed haplotype (Hap47), indicating possible historical connections with other regions.

The data obtained supplement existing knowledge about the epidemiology and pathogenesis of coenurosis in small ruminants, indicating the relevance of the problem for livestock farming in Kazakhstan. The results also emphasize the need to introduce molecular diagnostic methods into veterinary practice for early detection of the disease, effective control, and prevention of infection in herds. Overall, the results of the study confirm the importance of a comprehensive approach, including clinical, pathological, and molecular methods, for the diagnosis and study of coenurosis in sheep.

## Data Availability

The sequences used in this study have been deposited in the NCBI GenBank database under the following accession numbers: PP907960.1 – PP907963.

## References

[1] Rahsan Y Nihat Y Bestami Y Adnan A Nuran A. Histopathological, immunohistochemical, and parasitological studies on pathogenesis of *Coenurus cerebralis* in sheep. J Vet Res. (2018) 62:35–41. doi: 10.2478/jvetres-2018-0005, PMID: 29978125 PMC5957459

[2] Abera S Wubit A. Cerebral coenurosis in small ruminants: a review. J Anim Sci Adv. (2016) 6:1595. doi: 10.5455/jasa.20160409121545

[3] Li W-H Yang Y Zhang N-Z Wang J-K Liu Y-J Li L. Comparative transcriptome analyses of the developmental stages of Taenia multiceps. Front Vet Sci. (2021) 8:1–12. doi: 10.3389/fvets.2021.677045, PMID: 34291101 PMC8287169

[4] Ajaj EA Mohammad HA Gharban HAJ. First molecular confirmation of Coenurus cerebralis in sheep and goats with neurological behaviors in Iraq. Vet World. (2021) 14:1420–5. doi: 10.14202/vetworld.2021.1420-1425, PMID: 34316187 PMC8304432

[5] Dincel GC Yavuz O Yildirim S Al-Olayan EM El-Ashram S. ADAMTS-13 and HMGB1-induced oxidative stress in Taenia multiceps-infected animals. Sci Rep. (2023) 13:17929. doi: 10.1038/s41598-023-44376-0, PMID: 37863934 PMC10589341

[6] Guo C Xie Y Liu Y Wang N Zhan J Zhou X. Molecular characterization of Annexin B2, B3 and B12 in Taenia multiceps. Genes (Basel). (2018) 9:559. doi: 10.3390/genes9110559, PMID: 30463204 PMC6267623

[7] Liu Y Guo C Dong X Gu X Xie Y Lai W. Molecular characterisation and expression analysis of two heat-shock proteins in Taenia multiceps. Parasit Vectors. (2019) 12:93. doi: 10.1186/s13071-019-3352-8, PMID: 30867020 PMC6417115

[8] Akkus E Oguz F. Molecular prevalence of *Coenurus cerebralis* in sheep with neurological symptoms in Iğdır Province, Türkiye. Pak Vet J. (2024). 44:499–503. doi: 10.29261/pakvetj/2024.186

[9] Alvi MA Ohiolei JA Saqib M Tayyab MH Zafar Khan MU Li L. First report on molecular characterization of Taenia multiceps isolates from sheep and goats in Faisalabad, Pakistan. Front Vet Sci. (2020) 7:594–599. doi: 10.3389/fvets.2020.594599, PMID: 33240964 PMC7683608

[10] Zhang Y Zhao W Yang D Tian Y Zhang W Liu A. Genetic characterization of three mitochondrial gene sequences of goat/sheep-derived *coenurus cerebralis* and *cysticercus tenuicollis* isolates in Inner Mongolia, China. Parasite. (2018) 25:1–6. doi: 10.1051/parasite/2018002 PMC5774970 29350180

[11] Al Malki JS Hussien NA. Molecular characterization and phylogenetic studies of *Echinococcus granulosus* and *Taenia multiceps* coenurus cysts in slaughtered sheep in Saudi Arabia. Open Life Sci. (2021) 16:1252–60. doi: 10.1515/biol-2021-0131, PMID: 34901458 PMC8627918

[12] Abo-Shehada MN Jebreen E Arab B Mukbel R Torgerson PR. Prevalence of Taenia multiceps in sheep in northern Jordan. Prev Vet Med. (2002) 55:201–7. doi: 10.1016/S0167-5877(02)00056-9, PMID: 12383656

[13] Achenef M Markos T Feseha G Hibret A Tembely S. Coenurus cerebralis infection in Ethiopian highland sheep: incidence and observations on pathogenesis and clinical signs. Trop Anim Health Prod. (1999) 31:15–24. doi: 10.1023/A:1005125316275, PMID: 10399813

[14] Mohammed NH. Prevalence, morphological and biochemical study of larval stage *Coenurus cerebralis* of *Taenia multiceps* in sheep. Iraqi J Vet Sci. (2020) 34:159–63. doi: 10.33899/ijvs.2019.125660.1124

[15] Hajipour N Allah Rashidzadeh H Ketzis J Esmaeili seraji R Azizi H Karimi I. *Taenia ovis* in small ruminants in Iran: prevalence, pathology, and economic loss. Vet Sci. (2020) 7:34. doi: 10.3390/vetsci7010034, PMID: 32245116 PMC7158677

[16] Evangelisti MA Deiana R Melosu V Burrai GP Ballocco I Varcasia A. Relationships among neuroscore, magnetic resonance imaging features, and intracranial pressure in sheep affected by slow-growing brain lesions. Vet Radiol Ultrasound. (2018) 59:305–11. doi: 10.1111/vru.12589, PMID: 29274112

[17] Gazioglu A Simsek S Kizil O Ceribasi AO Kesik HK Ahmed H. Clinical, pathological and molecular evaluations and CT scan screening of coenurosis (Coenurus cerebralis) in sheep and calves. Rev Bras Parasitol Vet. (2017) 26:3–9. doi: 10.1590/s1984-29612016090, PMID: 28177040

[18] Carpio A Campoverde A Romo ML García L Piedra LM Pacurucu M. Validity of a PCR assay in CSF for the diagnosis of neurocysticercosis. Neurol Neuroimmunol Neuroinflamm. (2017) 4:e324. doi: 10.1212/NXI.0000000000000324, PMID: 28105460 PMC5241005

[19] Yera H Dupont D Houze S Ben M’Rad M Pilleux F Sulahian A. Confirmation and follow-up of neurocysticercosis by real-time PCR in cerebrospinal fluid samples of patients living in France. J Clin Microbiol. (2011) 49:4338–40. doi: 10.1128/JCM.05839-11 21976768 PMC3232946

[20] Amer S ElKhatam A Fukuda Y Bakr LI Zidan S Elsify A. Clinical, pathological, and molecular data concerning Coenurus cerebralis in sheep in Egypt. Data Brief. (2018) 16:1–9. doi: 10.1016/j.dib.2017.10.070, PMID: 29159244 PMC5684426

[21] Amrabadi O Oryan A Moazeni M Sharifiyazdi H Akbari M. Comparison of cerebral and non-cerebral coenurosis by genetic markers of glycolytic enzyme (enolase) and mitochondrial sequences in sheep and goats. Vet Parasitol. (2015) 214:333–6. doi: 10.1016/j.vetpar.2015.10.021, PMID: 26527237

[22] Kushaliyev K Ussenov Z Alimbekov S Mullakaev O Kozhayeva A Khairushev A. Study of the saiga helminth fauna and Ural sheep in the western region of Kazakhstan. Open Vet J. (2023) 13:485–94. doi: 10.5455/OVJ.2023.v13.i4.11, PMID: 37251266 PMC10219814

[23] Abbas I El-Alfy E-S Saleh S Tamponi C Varcasia A. Global epidemiology and molecular biology of *Taenia multiceps*: a comparative meta-analysis and *in silico* analysis study. Parasitology. (2022) 149:1607–22. doi: 10.1017/S0031182022001123, PMID: 35957580 PMC11010139

